# An intervention to control an ICU outbreak of carbapenem-resistant *Acinetobacter baumannii*: long-term impact for the ICU and hospital

**DOI:** 10.1186/s13054-018-2247-y

**Published:** 2018-11-21

**Authors:** Eli Ben-Chetrit, Yonit Wiener-Well, Emil Lesho, Puah Kopuit, Chaya Broyer, Liora Bier, Marc V. Assous, Shmuel Benenson, Matan J. Cohen, Patrick T. McGann, Erik Snesrud, Phillip D. Levin

**Affiliations:** 1 Infectious Diseases Unit, Shaare Zedek Medical Center, Hebrew University, Jerusalem, Israel; 2 Infectious Diseases Unit, Rochester Regional Health, Rochester, NY USA; 3 General Intensive Care Unit, Shaare Zedek Medical Center, Hebrew University, Jerusalem, Israel; 4 Clinical and Microbiology Laboratory, Shaare Zedek Medical Center, Hebrew University, Jerusalem, Israel; 5 Infectious Diseases Unit, Hadassah Hebrew University Medical Center, Jerusalem, Israel; 6 Clalit Health Services, Jerusalem, Israel; 7 Hebrew University, Jerusalem, Israel; 8 Multidrug-Resistant Organism Repository and Surveillance Network, Walter Reed Army Institute of Research, Silver Spring, MD USA

**Keywords:** *Acinetobacter baumannii*, ICU, Outbreak, Infection control

## Abstract

**Background:**

Following a fatal intensive care unit (ICU) outbreak of carbapenem-resistant *Acinetobacter baumanii* (CRAB) in 2015, an aggressive infection control intervention was instituted. We outline the intervention and long-term changes in the incidence and prevalence of CRAB.

**Methods:**

The infection control intervention included unit closure (3 days), environmental cleaning, hand hygiene interventions, and environmental culturing. CRAB acquisition and prevalence and colistin use were compared for the 1 year before and 2 years after the intervention.

**Results:**

Following the intervention, ICU CRAB acquisition decreased significantly from 54.6 (preintervention) to 1.9 (year 1) to 5.6 cases (year 2)/1000 admissions (*p* < 0.01 for comparisons with preintervention period.). Unexpectedly, ICU CRAB admission prevalence also decreased from 56.5 to 5.8 to 13 cases/1000 admissions (*p* < 0.001) despite the infection control intervention’s being directed at the ICU alone. In parallel, hospital CRAB prevalence decreased from 4.4 to 2.4 to 2.5 cases/1000 admissions (*p* < 0.001), possibly as a result of decreased discharge of CRAB carriers from the ICU to the wards (58.5 to 1.9 to 7.4 cases/1000 admissions; *p* < 0.001). ICU colistin consumption decreased from 200 to 132 to 75 defined daily dose (DDD)/1000 patient-days (*p* < 0.05). Hospital colistin consumption decreased from 21.2 to 19.4 to 14.1 DDD/1000 patient-days (*p* < 0.05).

**Conclusions:**

The ICU infection control intervention was highly effective, long-lasting, and associated with a decrease in last-line antibiotic use. The intervention was associated with the unexpected finding that hospital CRAB prevalence also decreased.

## Introduction

Carbapenem-resistant *Acinetobacter baumannii* (CRAB) has emerged globally as a significant and difficult-to-treat nosocomial pathogen among critically ill patients [1]. Numerous hospital outbreaks in intensive care units (ICUs) have been reported [2–5]. During the last decade, the prevalence of CRAB has increased worldwide [6]. Although previously considered a low-virulence pathogen [7, 8], its high attributable mortality has been well described [9, 10].

*Acinetobacter* outbreak termination is difficult to achieve, requiring patient screening and isolation strategies along with strict and Sisyphic environmental cleaning [6]. Temporary closure of the ICU may even be required [4]. In this report, we present the results of an intervention to terminate an outbreak of CRAB in the ICU, long-term follow-up, and associated changes in CRAB prevalence throughout the hospital.

## Methods

### Setting

The Shaare Zedek Medical Center is a 750-bed tertiary care teaching hospital in Jerusalem, Israel. During the period described, the ICU comprised 9 beds with approximately 500 adult surgical and medical admissions per year. During the study period, the ICU was open-plan with patient areas separated by curtains. The nursing ratio was one nurse to two patients. The ICU is a closed unit with full-time ICU physician coverage.

Prior to the intervention, 70% isopropyl alcohol-based hand rub together with single-use nonsterile gloves and gowns were available next to each patient. Chlorhexidine-based soap was available at five sinks. Continuous education of staff, as well as hand hygiene monitoring, compliance, and feedback, was routinely implemented.

Prior to the intervention, environmental cleaning was performed principally with 2000 ppm sodium hypochlorite. However, it was discovered that the solution was not prepared every day and that it was stored in an open container and thus susceptible to evaporation. Limited use was made of a quaternary ammonium-containing disinfectant (AntiGone; Teva Medical,  Modiin, Israel).

Throughout the study period (both before and after the intervention), perirectal surveillance swabs for carbapenem-resistant *Enterobacteriaceae* were obtained on ICU admission and weekly thereafter during ICU admission. Surveillance sputum cultures were obtained twice per week. Clinical cultures were obtained according to clinical indications. Standard protocols were used for central line insertion, maintenance, and removal and for prevention of central line-associated bloodstream infections and ventilator-associated infections.

### Outbreak description

CRAB was endemic in the hospital prior to 2015. Among the ICUs, almost the entire burden of CRAB colonization and infection was manifested in the general ICU. During the preintervention period, there were 57 CRAB cases in the general ICU, 8 in the cardiothoracic ICU, and 2 in the coronary care unit.

The mean daily hospital prevalence of CRAB during the preintervention period was lower than that of methicillin-resistant *Staphylococcus aureus* (MRSA) (mean number of cases per day, CRAB 12.3 ± 4.3 vs MRSA 15.1 ± 5.4, *p* < 0.001) but higher than that of carbapenem-resistant *Klebsiella pneumoniae* (3.1 ± 2.2 cases per day, *p* < 0.001 vs CRAB). It should be noted that extensive surveillance and cohorting were performed for *Klebsiella* cases but not for CRAB or MRSA cases.

A change in the clinical manifestation of CRAB bacteremia was noted during the first 5 months of 2015, with 11 CRAB patients with bacteremia developing catastrophic septic shock and multiorgan failure. Sources of bacteremia included seven cases of pneumonia, two cases of central line-associated bloodstream infection, and two others. Mortality was 82% within 72 h and 100% over 30 days. Despite reinforced infection control, environmental cultures (including bed rails, infusion pumps, computer keyboards) remained positive for CRAB even after terminal cleaning of patients’ units. The situation reached a crisis on 21 May 2015, when two ICU patients died simultaneously with CRAB bacteremia.

### Intervention

On 23 May 2015, an intervention team was assembled. It comprised senior hospital administrators, logistics representatives, ICU physicians and nurses, and infectious disease and infection control physicians. A control program was agreed upon and instituted on 25 May 2015. This included the following:

- Evacuation of the “infected” ICU with transfer of all patients to an alternative temporary ICU area. All existing equipment and stores from the “infected” ICU were either transferred with the patients or discarded.
- The empty “infected” ICU structure was cleaned using 2000 ppm sodium hypochlorite. A team of five cleaning staff working in shifts for 16 h per day was dedicated to the task for 3 days. After cleaning was completed, medical equipment not currently in use in the temporary ICU was cleaned and gradually transferred back. Adequacy of environmental cleaning was assessed with adenosine triphosphate (ATP) detection (ATP Complete® Contamination Monitoring System; Ruhof, Mineola, NY, USA) and surface microbiological culturing. ATP counts < 45 were considered to represent adequate cleaning. In most cases, postcleaning ATP counts were zero and never above the defined cutoff of 45. Results of all microbiological CRAB-selective cultures were negative.

**Fig. 1:**
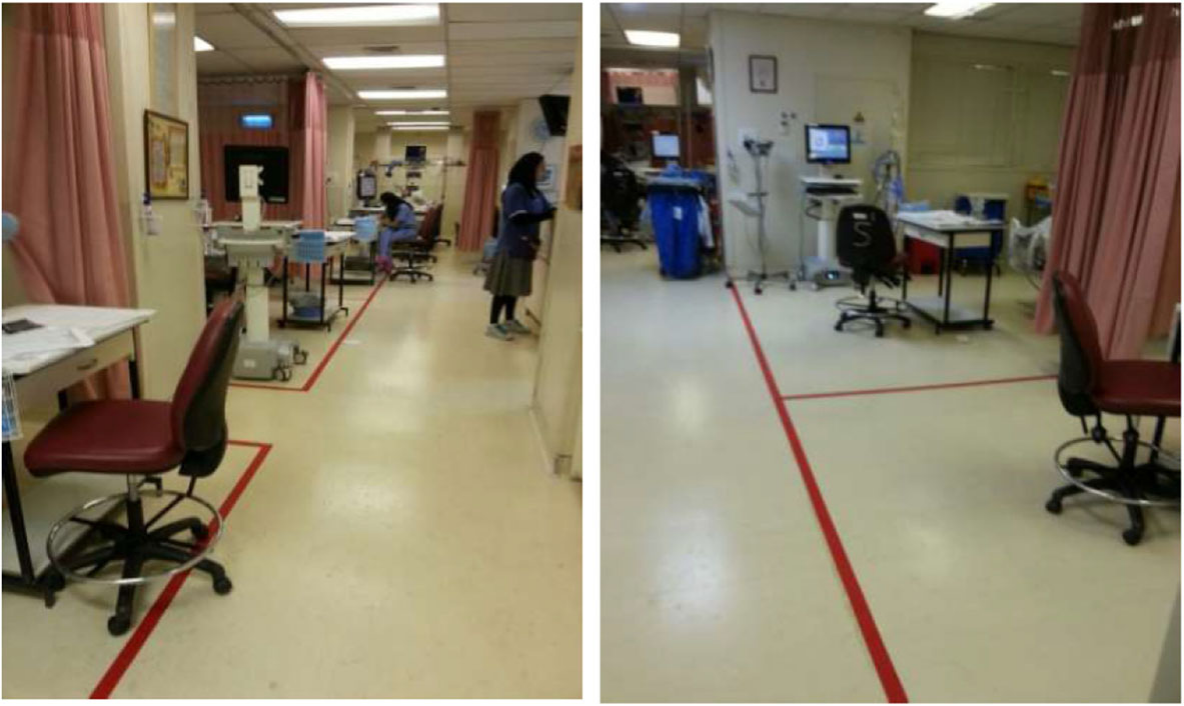
“Virtual walls” (*thick red lines*) in the open-plan intensive care unit

After 3 days, the “infected” ICU was declared CRAB-free, and the unit was replenished from the central hospital stores. Cleaned equipment was returned, and patients were readmitted as clinically indicated. The following steps were taken to prevent a recurrence:

1. In the absence of physical walls between patients, the boundary of each patient’s individual environment was defined by thick red lines painted on the floor (Fig. 1), defined as “virtual walls.” The staff was instructed that hand hygiene had to be performed when crossing a virtual wall in either direction. This instruction was reinforced by frequent verbal reminders. Within patient areas, gloves and gowns were required for any contact with the patient or bed. Contact with other patient equipment within the patient area (e.g., ventilator, pumps) did not require gown and gloves unless the patient was a carrier of a defined resistant organism (MRSA, vancomycin-resistant enterococci, carbapenemase-producing *Enterobacteriaceae*, or CRAB). Gloves, gowns, and alcohol-based hand sanitizers were positioned at the entrance to every patient area.
2. Use of shared medical equipment trolleys and shared portable computers was stopped. A computer was positioned in each defined patient area. Movement of equipment from one patient’s area to another was decreased by defining a patient area equipment list. Equipment was placed in each patient area according to the list prior to patient admission.
3. New cleaning personnel were employed, and protocols were devised for cleaning processes. A cleaning protocol was established that included the use of a new disposable cloth for each area, dating the 2000 ppm sodium hypochlorite solution, replacing it every 24 h and storing it in a closed container to prevent evaporation. Sensitive electronic equipment was cleaned using quaternary ammonium-containing wipes (AntiGone).
4. Hand hygiene observations and inspections by infection control nurses were significantly increased. In addition, educational sessions and reminders of standard precautions and hand hygiene practice were carried out by infection control physicians and nurses. Feedback was provided on hand hygiene compliance and colonization and infection rates. Environmental cultures and ATP measurements continued.
5. Screening cultures—rectal and sputum—were continued.
6. Specific treatment protocols (e.g., management of catheter-associated bloodstream infections) remained without change.
7. At the conclusion of the cleaning process, three ICU patients colonized with CRAB remained in the temporary ICU. These patients were transferred back to the original ICU but were cared for in a separate area, with separate equipment and stores. They were also treated by a dedicated nurse. After discharge of the last of these patients, the stores remaining in the “CRAB area” were discarded, and the equipment was cleaned and returned to the general ICU area. The “CRAB area” was thoroughly cleaned, checked using surface cultures and ATP, and then returned to general use.

### Microbiology

The environmental culturing technique was as follows: A 2″ × 2″ sterile gauze was wetted with 5 ml of a selective Mueller-Hinton broth containing vancomycin (6 g/l) and ertapenem (2 g/L). With use of sterile gloves, the sample area was swabbed with the wetted gauze. The gauze was then transferred to a sterile container with the addition of another 5 ml of culture broth. The container was maintained at room temperature for 72 h, and the broth was then plated for bacterial isolation [11]. Clinical cultures (blood, urine, sputum) and screening cultures (mainly rectal swabs) were processed and interpreted according to guidelines [12, 13]. Whole-genome sequencing of CRAB blood isolates from the preintervention period was performed to detect clonality and virulence genes using a MiSeq benchtop sequencer (Illumina Inc., San Diego, CA, USA) [14].

### Definitions and data analysis

1. ICU incident case (ICU acquisition): Negative ICU admission surveillance cultures, absence of CRAB in any cultures taken from 30 days prior until 48 h after ICU admission, but with CRAB in any culture taken thereafter in the ICU.
2. ICU prevalent case (admission prevalence): CRAB present in any culture taken during 30 days prior to ICU admission, in admission surveillance cultures, or in any cultures taken during the first 48 h of ICU admission.
3. Hospital prevalence: Patient-unique CRAB isolates from any culture taken outside the ICU. These cultures comprised principally clinical cultures because screening surveillance cultures were not obtained from patients outside the ICU.
4. Requirement for hand hygiene: Defined according to the World Health Organization “five moments” of hand hygiene [15].
5. ICU hand rub, gown and glove use: Determined by analysis of product release from central stores.
6. Antibiotic use: Defined daily dose (DDD) per 1000 patient-days calculated for colistin, meropenem, and piperacillin/tazobactam.

All data from the year prior to the intervention (1 June 2014 to 31 May 2015) were compared with each of the 2 years following the intervention (1 June 2015 to 31 May 2016 and 1 June 2016 to 31 May 2017). ICU CRAB incidence, admission prevalence, CRAB carriers discharged alive from the ICU, and hospital prevalence in adult medical and surgical wards were normalized to 1000 patient admissions. Use of colistin, meropenem, and piperacillin/tazobactam (DDD/1000 patient-days) was also compared.

In order to distinguish between the effects of the intervention and the decrease in ICU admission prevalence of CRAB on the risk of acquiring CRAB during ICU admission, two analyses were performed. First, ICU acquisition was normalized for admission prevalence, and second, a segmented regression analysis was performed using ICU incidence as the dependent variable and the intervention, ICU prevalence, and time as predictor variables [16–18].

Statistical analyses were performed using SAS 9.4 software (SAS Institute, Cary NC, USA). Student’s *t* test was used to compare continuous variables, and the chi-square test was performed for categorical variables. *p* < 0.05 was considered statistically significant. All tests were two-tailed.

A waiver of requirement for informed consent was approved by the hospital’s ethical review board (0036-17-SZMC).

## Results

ICU acquisition of CRAB decreased significantly from 54.6 cases/1000 admissions (28 cases) during the year prior to the intervention to 1.9 cases/1000 admissions (1 case) in the year following and to 5.6 cases/1000 admissions (3 patients) 2 years postintervention (*p* < 0.01 for comparisons with preintervention period). This decrease was accompanied by a significant decrease in ICU prevalence of CRAB (Table 1). The ICU patient admission characteristics (age, gender, Acute Physiology and Chronic Health Evaluation II score) remained constant, and the overall outcome of ICU patients in terms of ICU length of stay and mortality did not change (Table 1). Environmental cultures also showed improvement: Prior to the intervention, 5 of 14 (36%) environmental cultures were positive for CRAB vs 1 of 15 (7%) during the weeks immediately after the intervention (*p* = 0.025).

**Table 1:** Demographics and *Acinetobacter baumanii* prevalence in the intensive care unit and hospital before and after intervention (cases per 1000 admissions)

	Before intervention (period 1)	One year postintervention (period 2)	Two years postintervention (period 3)
ICU admissions (*n*)	513	516	537
Age (years)	61 ± 20.2	50 ± 21.7	59 ± 21.2
Gender (male)	277 (54%)	300 (58.1%)	306 (57%)
APACHE II score	18.3 ± 8.1	18.4 ± 8.1	18.3 ± 8.0
Median ICU length of stay (days)	3 (2–6)	3 (2–7)	3 (2–6)
ICU mortality	58 (11.3%)	52 (10%)	50 (9%)
CRAB patients			
CRAB ICU acquisition^a^	54.6 (*n* = 28)	1.9 (n = 1)^b^	5.6 (*n* = 3)^b^
CRAB carriers discharged alive from ICU to hospital wards^a^	58.5 (*n* = 30)	1.9 (n = 1)^b^	7.4 (*n* = 4)^b^
CRAB ICU admission prevalence^a^	56.5 (*n* = 29)	5.8 (n = 3)^b^	13.0 (*n* = 7)^b^
Median ICU length of stay (days)	13 (5–22)	7 (3–28)^c^	
Median time from ICU admission until CRAB acquisition (days)	7 (4–11)	4 (3–32)^c^	
CRAB hospital mortality	31/57 (54%)	4/4 (100%)	6/10 (60%)
Medical and surgical wards			
Admissions (*n*)	39,444	41,006	44,113
Hospital wards CRAB prevalence^a^ (clinical cultures)	4.4 (*n* = 173)	2.4 (*n* = 99)^b^	2.5 (*n* = 111)^b^

*Abbreviations: APACHE II* Acute Physiology and Chronic Health Evaluation II, *CRAB* Carbapenem-resistant *Acinetobacter baumanii*, *ICU* Intensive care unit

^a^Cases/1000 admissions

^b^*p* < 0.001 for comparison with preintervention period

^c^One and two years postintervention combined owing to small numbers, *p* = ns compared to preintervention period

**Fig. 2:**
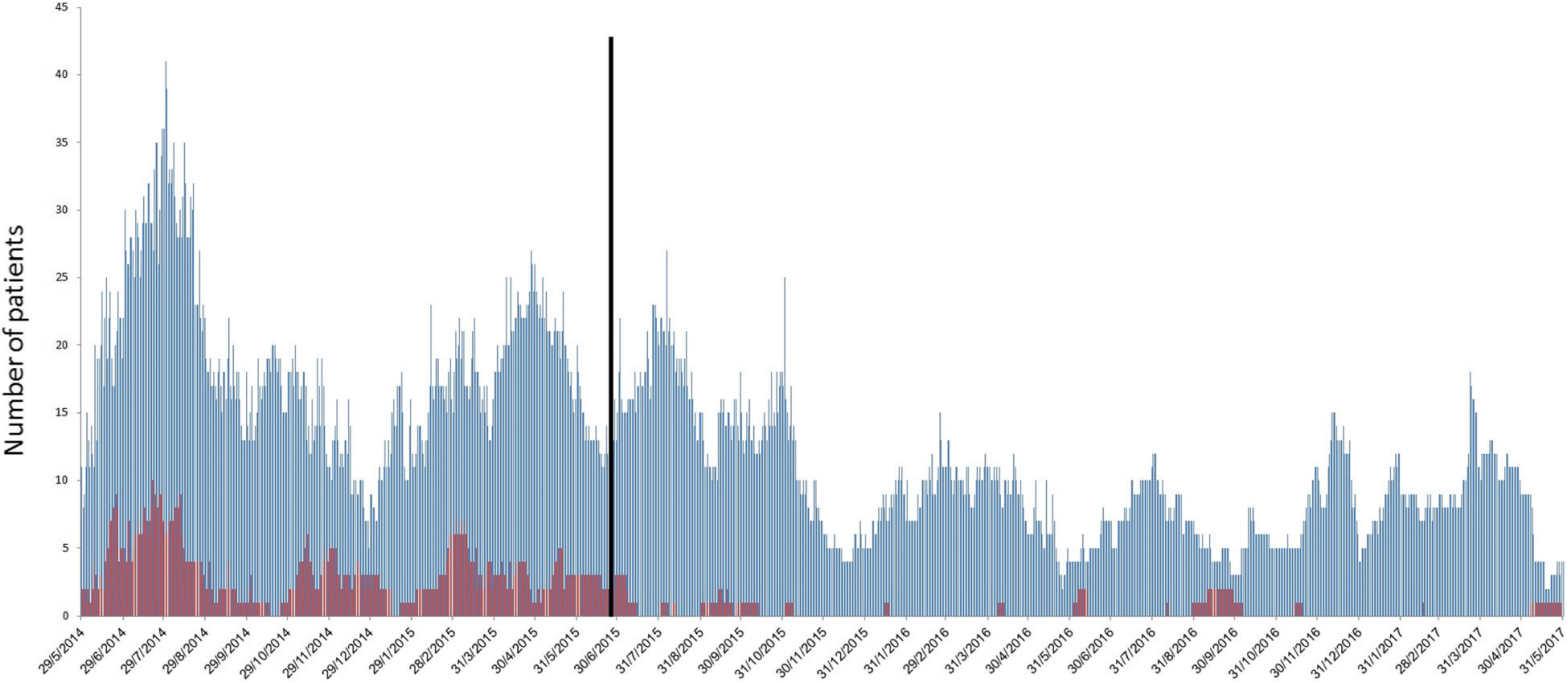
Carbapenem-resistant *Acinetobacter baumanii* daily prevalence in the intensive care unit (*red bars*) and in the medical and surgical departments (*blue bars*). The *vertical line* represents the time point of intervention

Unexpectedly, these changes were also accompanied by a decrease in the ICU admission prevalence of CRAB (from a total of 29 patients/year preintervention to 3 and 7 patients/year, 1 and 2 years postintervention, respectively; *p* < 0.001 for comparisons with preintervention period). A significant decrease in hospital prevalence of CRAB was also noted (Table 1 and Fig. 2). In an attempt to identify the cause of the hospital-wide decrease in CRAB, the number of CRAB-positive carriers discharged from the ICU to the hospital wards was assessed and found to have decreased significantly (from a total of 30 patients/year preintervention to 1 and 4 patients/year 1 and 2 years postintervention, respectively; *p* < 0.001 for comparisons with preintervention period).

In order to distinguish between the effects of the intervention itself and the decrease in ICU CRAB prevalence on CRAB acquisition in the ICU, two further analyses were performed. First, ICU CRAB acquisition was normalized for ICU admission prevalence: preintervention there were 28 acquisitions/29 admission prevalent cases = 0.975 acquisitions per prevalent admission vs 4/10 = 0.4 postintervention (*p* < 0.001). Second, a segmented regression analysis was performed using ICU CRAB acquisition as the dependent variable and the intervention, total ICU prevalence (not only admission prevalence), and time as predictor variables. Both the intervention and ICU prevalence were significant predictors of ICU acquisition, but in opposite directions (intervention: beta = − 0.081; 95% CI, − 0.131, − 0.031; *p* = 0.001; ICU prevalence: beta = 0.045; 95% CI, 0.033, 0.058; *p* < 0.001). In other words, the intervention was associated with a significant decrease in the risk of ICU CRAB acquisition that was approximately twice the decrease in risk of acquisition associated with lower prevalence.

**Fig. 3:**
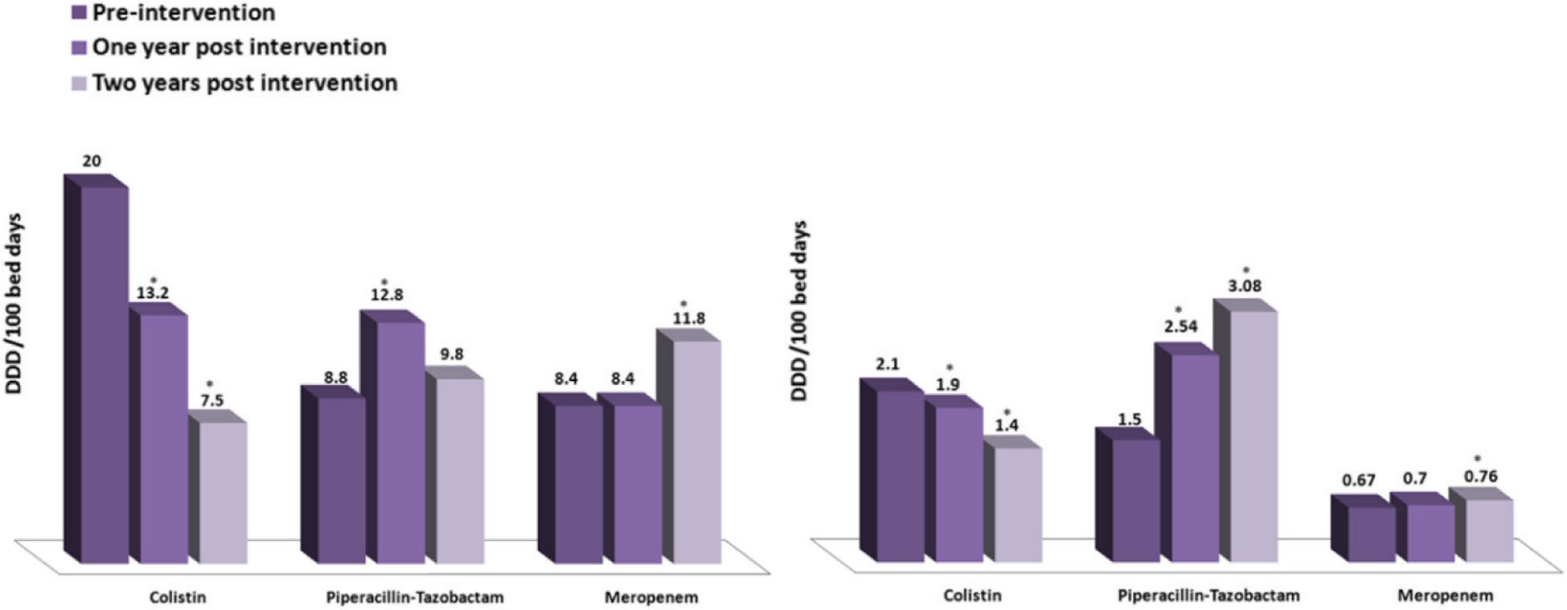
Colistin, piperacillin-tazobactam, and meropenem consumption (defined daily dose/100 bed days) before and after intervention in the intensive care unit (left panel) and in the medical and surgical departments (right panel). *p < 0.05 for comparison with preintervention period

Comparison of antibiotic use in the ICU before and after the intervention showed a significant decrease in the use of colistin, no change in use of meropenem, and a significant increase in use of piperacillin-tazobactam during the first year postintervention. Similar changes were found outside the ICU (Fig. 3).

The number of ICU hand hygiene compliance observations performed by the infection control nurses increased from 737 prior to intervention to 1700 one year postintervention and to 2940 during the second year postintervention, with an increase in compliance from 84% to 93% and 97%, respectively (*p* < 0.001 for comparisons with preintervention period). A concordant significant increase in consumption of isopropyl alcohol-based hand sanitizer (a proxy measure of hand hygiene performance that did not measure quality or timing of hand hygiene performance) was recorded (91 ± 20 vs 184 ± 40 vs 206 ± 29 bottles per month for 2014, 2015, and 2016, respectively; *p* < 0.001 for 2015 vs 2014 and 2016 vs 2015).

Whole-genome sequencing of CRAB blood isolates revealed that the outbreak consisted of two distinct bacterial clusters. Cluster 1 was composed of seven strains belonging to MLST-3 (Multilocus Sequence Typing ST-3, clonal complex 3). The strains were separated by 1 to > 400 single-nucleotide polymorphisms (SNPs) and could be further divided into 4 closely related but distinct populations. Cluster 2 consisted of four strains belonging to MLST-2 (ST-2, clonal complex 2). The strains were separated by 14–107 SNPs and likely represent distinct populations. None of the strains were related to the highly virulent ST-10 group.

## Discussion

We describe a highly successful intervention to terminate an aggressive CRAB outbreak in our ICU. The intervention reduced ICU CRAB acquisition almost to zero, a change that has been maintained for over 2 years. The intervention was associated with a significant decrease in colistin use. The intervention was also associated with a general decrease in CRAB prevalence outside the ICU, possibly suggesting that the ICU represents an engine for CRAB propagation within the hospital.

Preventing spread of infection within an ICU is challenging. Prevention is based on elements common to the whole ICU, elements specific to particular patients (such as neutropenic patients, patients with burns), particular pathogens (such as *Clostridium difficile* or multidrug-resistant bacteria), particular infections (such as tuberculosis, respiratory or other airborne viruses), and elements specific to particular interventions (such as prevention of ventilator-related infections or central line-associated infections). The common elements are based principally on preventing spread from patients to patients and reducing pathogen load in the environment. These interventions are particularly relevant for *Acinetobacter*, a bacterium that is long-lasting on environmental surfaces (i.e., has a large environmental reservoir) and is highly efficient at passing from patient to patient. Our intervention was aimed principally at the common elements, which, although simple in principle, require attention to endless detail [19].

The infection control intervention employed was based on accepted tenets of unit closure, intense environmental cleaning, environmental cultures, and improved infection prevention practices by the staff [3, 4, 20, 21]. Examination of the cleaning processes lead to the discovery of several loopholes, such as undated hypochlorite solutions, maintenance of hypochlorite solutions in open containers (with the risk of evaporation), use of nondisposable cleaning cloths in multiple patient areas, and others. The environmental cultures provided important feedback for the ICU and cleaning staff. The loopholes were closed with education, checklists, and follow-up cultures. Hand hygiene compliance was also not optimal.

In addition, “virtual walls” were instituted. Several reports have suggested that the use of single-patient rooms is associated with a decrease in the risk of acquiring resistant pathogens [22–25]; however, our ICU was open-plan. In an open-plan unit, the precise moment that hand hygiene is required when moving from one patient’s area to the next can be difficult to identify for the ICU staff. By marking thick red lines on the floor between patients’ areas (Fig. 1), the “virtual wall” clearly defined when hand hygiene had to be performed. The presence of a binary decision (crossing the red line) facilitated education, measurement, and recommendations for compliance. Since then, the virtual walls have been introduced in several other hospital areas with equal success.

As the postintervention period continued, we were surprised to find that fewer and fewer CRAB-positive patients were being admitted into the ICU. This was unexpected because the intervention was limited to the ICU and the prevalence of CRAB in the hospital wards was not expected to change. Examination of the data revealed that the number of ICU patients leaving the ICU alive while colonized with CRAB had decreased from 48 patients/year to 1 patient/year, presumably as a result of decreased acquisition in the ICU. The decrease in CRAB prevalence outside the ICU (from 185 to 101 patients/year, a difference of 84 patients) exceeded the decrease in CRAB “export” from the ICU (47 patients). This could suggest that each CRAB patient that leaves the ICU represents a focus of infection leading to spread in the hospital wards.

As CRAB became a less frequent pathogen in the ICU and hospital, so empiric colistin use decreased. This represented an important clinical gain in terms of antibiotic stewardship.

The bacterial genome was assessed in order to determine whether the change in CRAB behavior during 2015 (with an increase in the incidence of catastrophic septic shock) represented spread of a new hypervirulent clone. Recently, Jones et al. reported a fatal CRAB outbreak in immunocompetent patients caused by an extensively drug-resistant and virulent CRAB strain identified as the clade B strain, belonging to ST-10 group [10]. Whole-genome sampling of the CRAB isolates from the fatal bacteremia cases in our unit revealed that none belonged to this highly virulent strain.

**Fig. 4:**
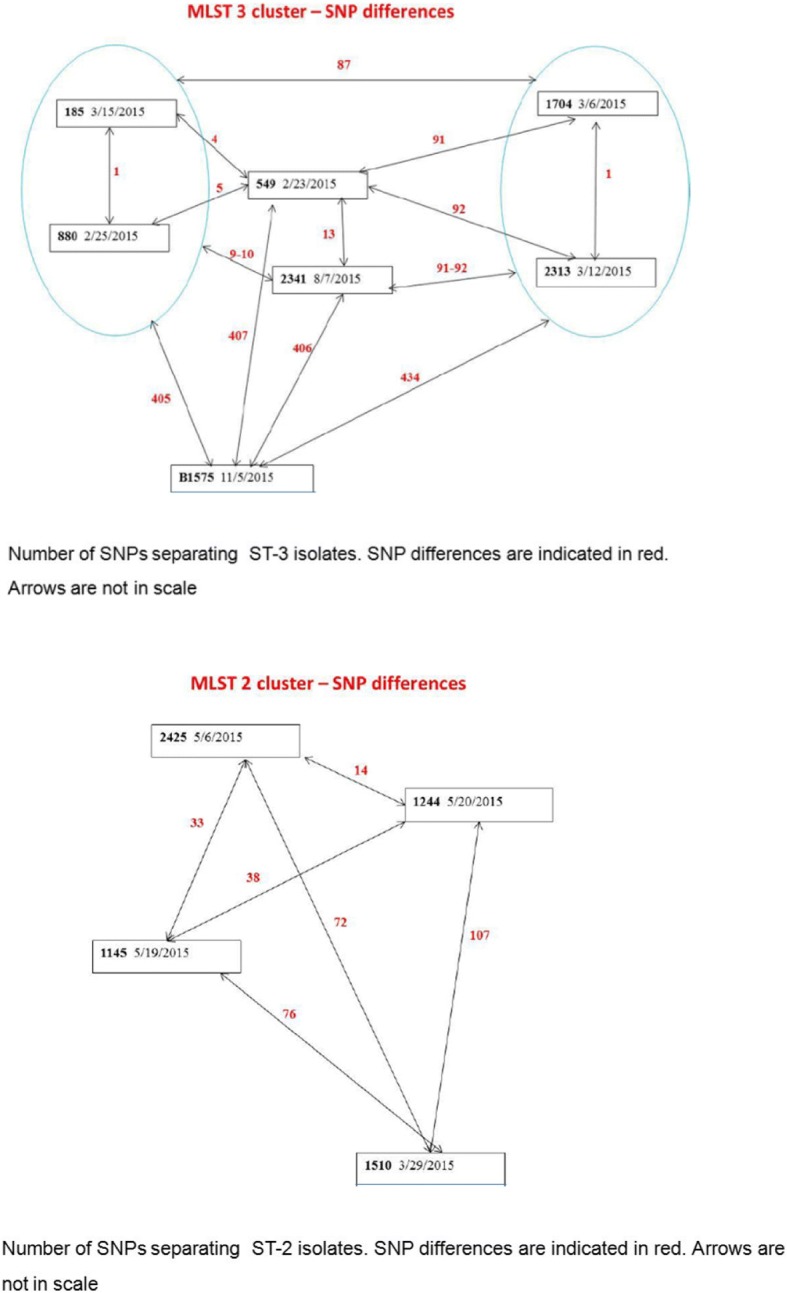
MLST (Multilocus Sequence Typing) cluster analysis and single-nucleotide polymorphism differences between carbapenem-resistant *Acinetobacter baumanii* blood isolates

The whole-genome sampling did, however, shed light onto the spread of CRAB in the ICU. Whole-genome sampling is a useful tool to determine whether bacteria infecting different patients result from transmission from patient to patient (where genetic similarity is high) or represent infection from separate sources (where genetic similarity is low). Genetic homogeneity is measured by cluster similarity and SNPs. For CRAB, there are two common global clusters that cause infections in hospital patients: MLST2 and MLST3. Both were identified in our ICU during overlapping time periods. Regarding SNP diversity, in order to determine that the same bacteria caused infection in two patients (resulting from patient-to-patient transfer), an SNP difference of < 4 base pairs should be identified. For CRAB, a further complication is spontaneous genetic change or the molecular clock, which predicts that 10–15 SNPs will change per year within a bacterial clone. Taking into account these considerations, the infections caused by bacteria in the MLST2 cluster probably all came from different sources. The closest genetic similarity in the cluster was a difference of 14 SNPs (patients 2425 and 1244) (Fig. 4) that could represent infection from a common source that had persisted in the environment for over 1 year. In contrast, several bacteria isolated from in the MLST3 cluster had very high genetic similarity. Two pairs of patients had bacteria with only one SNP difference (implying patient-to-patient transmission within the ICU), and a fifth patient had a bacteria with a four-SNP difference (pair 1: patients 1704 and 2313, pair 2: 185 and 880 similar to patient 549) (Fig. 4). The implications of these findings are that prior to the intervention there may have been untreated environmental reservoirs of CRAB within the ICU, repeated import of CRAB into the ICU (either with admission of new patients, on fomites, or by staff), and patient-to-patient transmission within the ICU [6, 26–28].

Our investigation has several limitations:

1. It is a single-center trial.
2. The causes of an outbreak and of its resolution are often difficult to elucidate, and furthermore, the before-after description of the intervention cannot prove causality; it can only describe associations. Despite this, the close temporal relationship of the intervention to the decline in CRAB incidence and prevalence, both in the ICU and outside it, and prolonged follow-up for 2 years may suggest a causative effect.
3. We were unable to assess incident CRAB cases outside the ICU because surveillance sampling is not performed outside the ICU routinely. This may also have led to underestimation of CRAB prevalence in the hospital wards. Culturing practices did not change before and after the intervention, however, meaning that the data should have been comparable.
4. It is possible that the intervention in the ICU had an educational impact outside the ICU that caused the decline in CRAB prevalence.
5. In terms of quantitative observations of specific infection prevention interventions, only data on hand hygiene observations, alcohol hand rub use, and environmental cultures were available from before and after the intervention.

## Conclusions

We present the success of an aggressive and multifaceted intervention in controlling a CRAB outbreak in the ICU and its unexpected association with decreased CRAB prevalence at ICU admission and in the hospital wards outside the ICU.

## Abbreviations

APACHE II: Acute Physiology and Chronic Health Evaluation
ATP: Adenosine triphosphate
CRAB: Carbapenem-resistant *Acinetobacter baumannii*
DDD: Defined daily dose
ICU: Intensive care unit
MLST: Multilocus Sequence Typing
MRSA: Methicillin-resistant *Staphylococcus aureus*
SNP: Single-nucleotide polymorphism
